# Risk Factors For Radiation-Induced Skin Ulceration in Percutaneous Coronary Interventions of Chronic Total Occluded Lesions: A 2-Year Observational Study

**DOI:** 10.1038/s41598-017-08945-4

**Published:** 2017-08-16

**Authors:** Chi-Cheng Lai, Kai-Che Wei, Wen-Yee Chen, Guang-Yuan Mar, Wen-Hwa Wang, Chieh-Shan Wu, Ching-Jiunn Tseng, Kuo-Chung Yang, Lee-Wei Chen, Chun-Peng Liu

**Affiliations:** 1 Cardiovascular Center, Kaohsiung Veterans General Hospital, Kaohsiung, Taiwan; 2 Department of Internal Medicine, Tri-Service General hospital, National Defense Medical Center, Taipei, Taiwan; 3 School of Medicine, National Yang-Ming University, Taipei, Taiwan; 4 Department of Biological Sciences, National Sun Yat-Sen University, Kaohsiung, Taiwan; 5 Department of Dermatology, Kaohsiung Veterans General Hospital, Kaohsiung, Taiwan; 6 Faculty of Yuhing Junior College of Health Care and Management, Kaohsiung, Taiwan; 7 Department of Emergency, Kaohsiung Veterans General Hospital, Kaohsiung, Taiwan; 8 College of Health and Nursing, Meiho University, Pingtung, Taiwan; 9 Department of Medical Education and Research, Kaohsiung Veterans General Hospital, Kaohsiung, Taiwan; 10 Division of Plastic and Reconstructive Surgery, Kaohsiung Veterans General Hospital, Kaohsiung, Taiwan; 11 Department of administration, Kaohsiung Veterans General Hospital, Kaohsiung, Taiwan

## Abstract

Relationship between radiation-induced skin ulceration (RSU) and variables in percutaneous coronary interventions (PCI) was rarely reported. RSU is a severe complication in PCIs, especially for chronic total occlusion (CTO) lesions. We investigated the RSUs and their risk factors in patients receiving CTO PCIs over a 2-year period. Data were analyzed using chi-square tests, t-tests and receiver operating characteristic (ROC) curve. Of 238 patients, 11 patients (4.6%) had RSUs all at right upper back. RSUs were significantly associated with use of left anterior oblique (LAO) views (100% vs. 47.1%, p < 0.001), retrograde techniques (36.3% vs. 7.9%, p = 0.012), or a procedure time (PT) defined as a time duration between the first and last angiograms of > 120, 180, or 240 minutes (p < 0.05). ROC analysis showed a long PT was an accurate predictor of RSUs (AUC = 0.88; p < 0.001) at a cut-off of 130 minutes (sensitivity = 0.91, specificity = 0.81). The results showed risk factors for RSUs containing use of large LAO views, retrograde techniques, and prolonged PTs. This study suggests that, to minimize RSU, interventionalists should limit PT to roughly 2 hours in fixed LAO views.

## Introduction

Coronary artery disease (CAD) is one of the leading causes of mortality globally^1^. Percutaneous coronary intervention (PCI) is an option for treating CAD. As the aged population grows in many countries, the prevalence of CAD and the complexity of PCI both increase proportionally. Complex PCIs, particularly in patients with chronic total occlusion (CTO) lesions, are generally associated with increased procedure times (PTs), radiation exposure, and complications^2, 3^. Although successful PCIs for CTO lesions may improve long-term survival^4^, such complex PCIs with prolonged radiation exposure potentially cause radiation-induced skin injury. Radiation-induced skin ulceration (RSU) is a rare^5–8^ but severe skin complication^8, 9^. RSUs always require surgical treatments^8^. Therefore, minimising radiation-induced skin complications has emerged as a crucial task for coronary interventionalists. Primarily, elucidating the risk factors for radiation-induced skin injury is essential. Real-time risk estimates of RSU events appear essential to minimise the development of RSU complications during prolonged PCIs^9–12^. Relevant literature is scant, with only sporadic case reports of the relationship between radiation-induced skin complications and technical parameters^5–8^. The present observational study is the first investigation of RSU events and their relationship with PCI-related variables in patients who received at least one index CTO PCI. The aims of the study were (1) to select patients with and without RSUs after CTO PCIs and investigate the presentations of RSUs over a 2-year study period; (2) to elucidate PCI-related risk factors for RSU events; and (3) to identify an intraprocedural indicator to facilitate coronary interventionalists in reducing RSU events.

## Results

### Baseline characteristics

**Figure 1:**
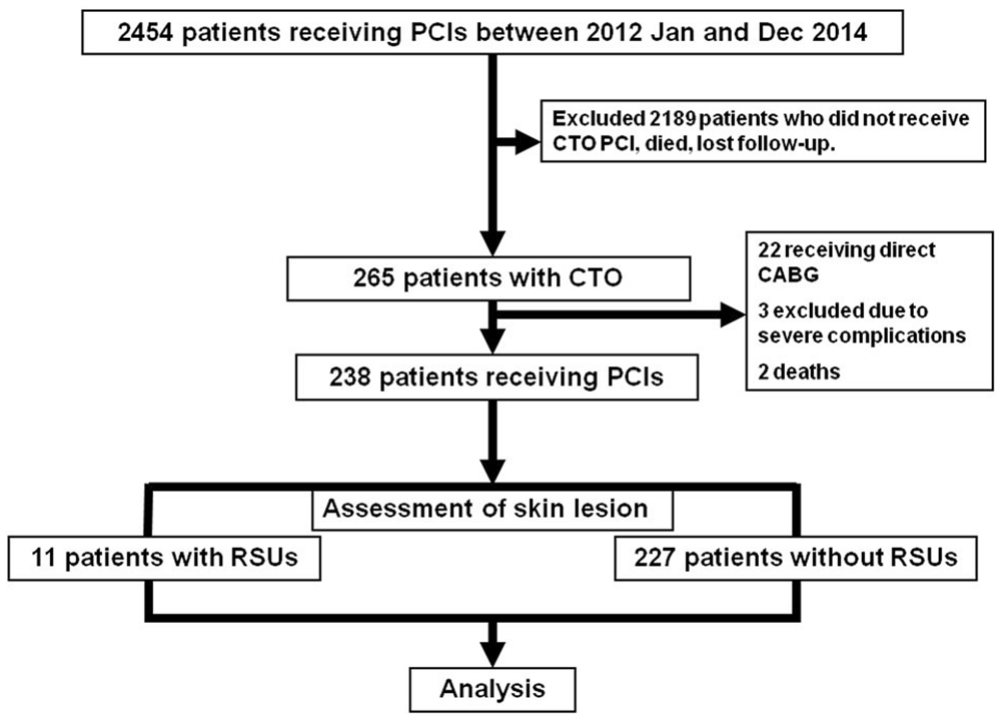
Patient flow.

This study analysed 238 patients who had received at least one CTO PCI between 1 January 2012, and 31 December 2013. Of the 238 patients, 11 patients (4.6%) had RSUs, whereas 227 patients (95.4%) did not. The patient flow is outlined in Fig. 1. The baseline characteristics and PCI-related variables of the patients are listed in Table 1.

**Table 1:** Baseline Characteristics in Patients Receiving CTO PCIs.

	Patients (n = 238)
Age (years)	64.4 ± 13.7
Gender (male/female)	216/22
Hypertension	176 (73.9)
Diabetes mellitus	93 (38.9)
Dyslipidemia	131 (55.0)
Prior CABG	21 (8.8)
Approach	
TRA	96 (40.3)
TFA	69 (29.0)
TFA + TFA	31 (13.0)
TRA + TFA	41 (17.2)
Others	1 (0.4)
Guiding catheter technique	
Single GC	75 (31.5)
Double GC	163 (68.5)
CAD lesions	
Single vessel disease	25 (10.5)
Two vessel disease	69 (28.9)
Three vessel disease	113 (47.3)
Others^a^	32 (13.4)
Target vessel for CTO PCI	
RCA	110 (46.2)
LAD	72 (30.3)
LCX	53 (22.3)
Others^b^	3 (1.3)
Approach	
Antegrade (no retrograde)	216 (90.8)
Retrograde (plus antegrade)	22 (9.2)
Procedure time^c^	94.9 ± 66.4
Technical results	
Success^d^	185 (77.7)
Failure	53 (22.3)
RSU cases (n, %)	11 (4.6)
Location	
Right scapular or para-scapular area	11 (100)

Values are means ± standard deviation or n (%).

CTO = chronic total occlusion; PCI = percutaneous coronary intervention; CABG = coronary artery bypass grafting; TRA = trans-radial artery approach; TFA = trans-femoral artery approach; GC = guiding catheter; CAD = coronary artery disease; RCA = right coronary artery; LAD = left anterior descending artery; LCX = left circumflex artery; RSU = radiation skin ulcer.

^a^Involvement of left main, left internal mammary artery, or greater saphenous vein.

^b^PCI at multiple CTO vessels.

^c^Procedure time was defined as the interval between the first and last angiograms.

^d^Completion of implantation of coronary stent(s).

### RSU lesions

**Figure 2:**
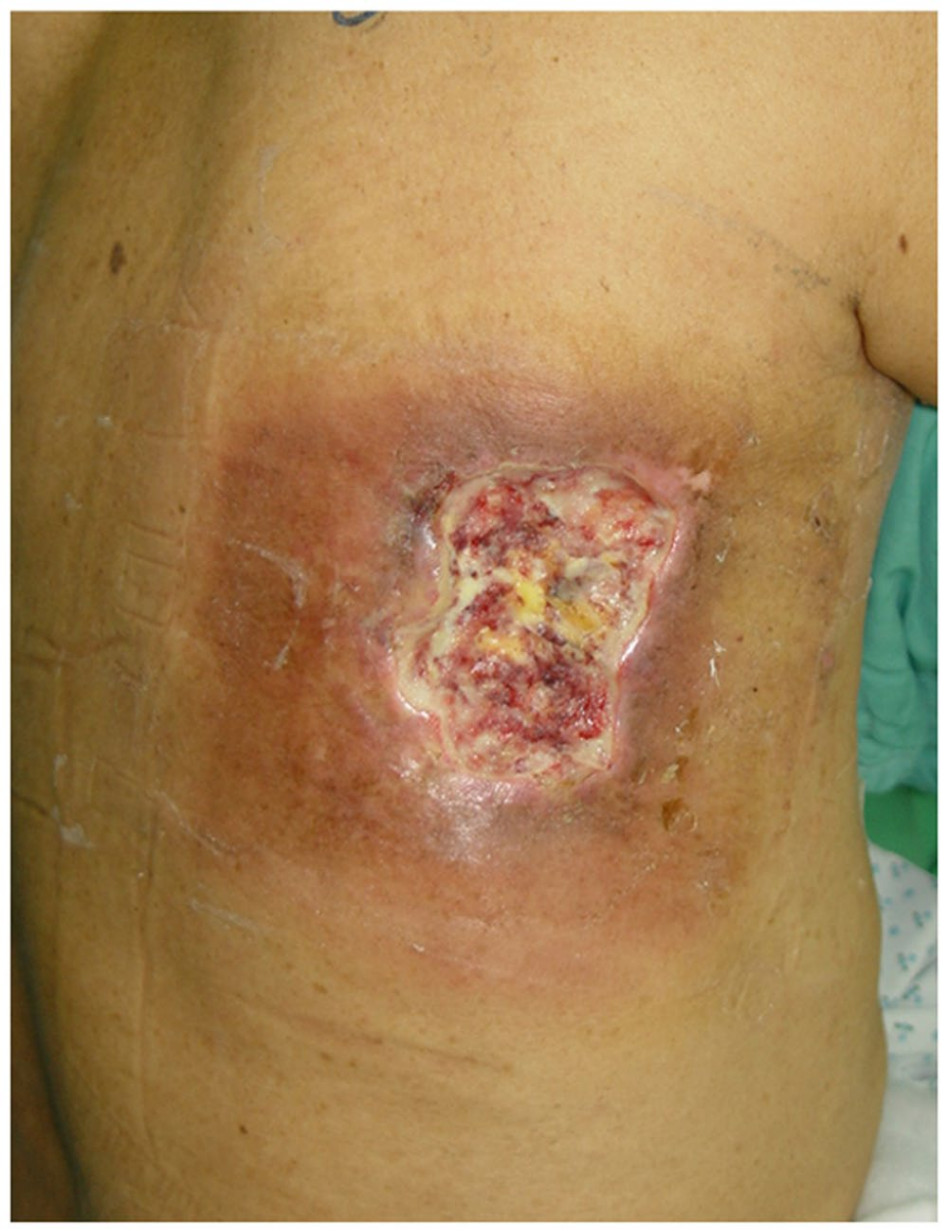
A RSU case before surgery is shown. Square-shaped sharply-demarcated erythematous-to-brownish patch with a central ulcer is displayed in the right subscapular area of an 81-year-old male at 7 months after undergoing a prolonged percutaneous coronary intervention for chronic total occluded lesions.

**Figure 3:**
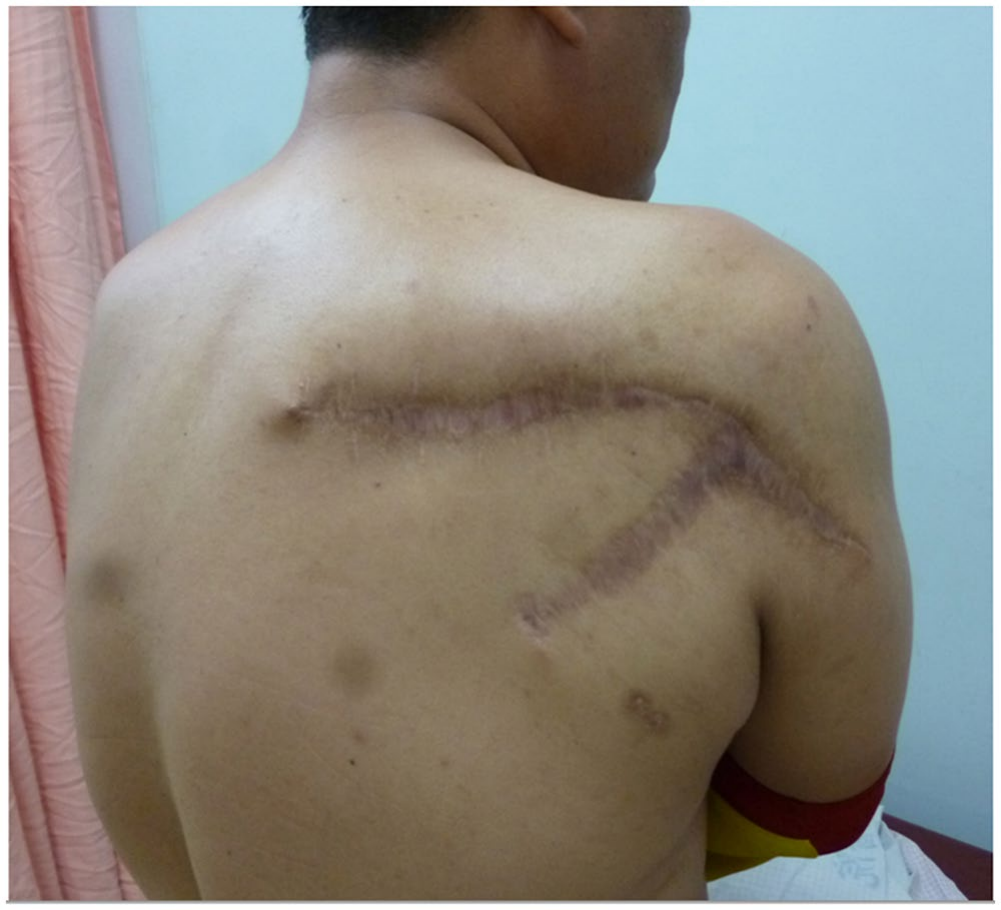
A RSU case after surgery is shown. Radiation-induced skin ulceration in a 43-year-old male was treated by radical excision of the ulcer wound and reconstruction with rotation flap. Skin healing progress was good at 10 months after plastic surgery.

The RSU lesions gradually developed within several weeks or months after the index CTO PCIs. All RSU lesions were located on the right upper back (11/11, 100%), and some extended to the right upper arm. A typical RSU lesion is shown in Fig. 2. The lesion had a clear margin with surrounding hyperpigmentation, a relatively pale centre, and poorly healing core wounds. All RSU lesions ultimately required plastic surgery, including debridement, skin graft, or flap reconstruction (11/11, 100%). A wound scar after surgical treatment of an RSU lesion is displayed in Fig. 3.

### RSU and PCI parameters

Compared with the patients without RSUs, a greater rate of prior PCI (100% vs. 26.0%, p < 0.001); a higher number of PCIs of the non-left anterior descending (LAD) arteries (non-LAD arteries including the right coronary arteries [RCA] or left circumflex arteries [LCX]; 100% vs. 31.7%, p = 0.037); more frequent use of large left anterior oblique (LAO) angles (100% vs. 47.1%, p < 0.001); more frequent use of retrograde approaches (36.3% vs. 7.9%, p = 0.012); greater estimated fluoroscopy times (136.5 ± 69.7 vs. 48.3 ± 33.1 minutes, p < 0.001); and longer PTs >1.5 hours (90.9% vs. 37.4%, p = 0.001), >2 hours (90.9% vs. 24.4%, p = 0.001), >2.5 hours (81.8% vs. 12.8%, p < 0.001), >3 hours (72.7% vs. 7.0%, p < 0.001), >3.5 hours (45.5% vs. 4.0%, p < 0.001), and >4 hours (18.2% vs. 1.8%, p = 0.026) were observed for patients with RSUs. In addition, compared with those without RSUs, patients with RSUs had a significantly longer PT (226.5 ± 139.2 vs. 88.5 ± 53.7 minutes, p = 0.008). The occurrence of RSU events was not statistically related to sex; age; history of diabetes mellitus, hypertension, or dyslipidemia; history of coronary artery bypass grafting; arterial approach; or technical success or not (all p > 0.05) (Table 2).

**Table 2:** Data Comparison Between Patients With and Without RSU

	Patients without RSU (n = 227)	Patients with RSU (n = 11)	p value
Male (n)	205 (90.3)	11 (100)	0.606
Age (years)	64.6 ± 13.7	60.8 ± 13.8	0.243
Hypertension	167 (73.6)	9 (81.8)	0.733
Diabetes mellitus	86 (37.9)	7 (63.6)	0.115
Dyslipidemia	122 (53.7)	9 (81.8)	0.117
History of CABG	20 (8.8)	1 (9.1)	1.000
Prior PCI	59 (26.0)	11 (100)	<0.001
Variables in index PCI			
No. of diseased vessel			0.253
Single vessel disease	22 (9.7)	3 (27.3)	
Double vessel disease	67 (29.5)	2 (18.2)	
Three vessel disease	107 (47.1)	6 (54.5)	
Others^a^	31 (13.7)	1 (9.1)	
Approach			0.253
TRA	93 (41.0)	3 (27.3)	
TFA	63 (27.8)	5 (45.5)	
TRA plus TFA	41 (18.1)	0 (0)	
TFA plus TFA	29 (12.8)	3 (27.3)	
Others^b^	1 (0.4)	0 (0)	
GC technique			
Single/Double (n/n)	156/71	7/4	0.745
Single target CTO vessel			0.006
RCA	84 (37.0)	7 (63.6)	
LAD	65 (28.6)	0 (0)	
LCX	27 (11.9)	4 (36.4)	
Others^c^	51 (22.5)	0 (0)	
RCA vs. Non-RCA PCI	103 (45.4)	7 (63.6)	0.354
LAD vs. Non-LAD PCI	72 (31.7)	0 (0)	0.037
LCX vs. Non-LCX PCI	49 (21.6)	4 (36.4)	0.269
Main use of a large LAO view	107 (47.1)	11 (100)	<0.001
PCI strategy (n/n)			
Retrograde/Non-retrograde	18/209	4/7	0.012
Fluoroscopic time^d^ (minutes)	48.3 ± 33.1	136.5 ± 69.7	<0.001
PT (hours)			
>1	143 (63.0)	10 (90.9)	0.103
>1.5	85 (37.4)	10 (90.9)	0.001
>2	55 (24.4)	10 (90.9)	<0.001
>2.5	29 (12.8)	9 (81.8)	<0.001
>3	16 (7.0)	8 (72.7)	<0.001
>3.5	9 (4.0)	5 (45.5)	<0.001
>4	4 (1.8)	2 (18.2)	0.026
Mean (minutes)	88.5 ± 53.7	226.5 ± 139.2	<0.001^e^
Technical success^f^	179 (78.9)	6 (54.5)	0.071

Values are means ± standard deviation or n (%).

PCI = percutaneous coronary intervention; RSU = radiation-induced skin ulcer; CABG = coronary artery bypass grafting; TFA = trans-femoral artery approach; TRA = trans-radial artery approach. GC = guiding catheter; CTO = chronic total occlusion; RCA = right coronary artery; LAD = left anterior descending artery; LCX = left circumflex artery; LAO = left anterior oblique; PT = procedure time.

^a^Involvement of left main, left internal mammary artery, or greater saphenous vein.

^b^Trans-brachial artery approach.

^c^PCI at multiple CTO vessels or left main bifurcation.

^d^A fluoroscopy time was estimated for each index CTO PCI.

^e^Analysis using Mann-Whitney *U* test.

^f^Completion of implantation of coronary stent(s).

### Risk factors for RSU

**Figure 4:**
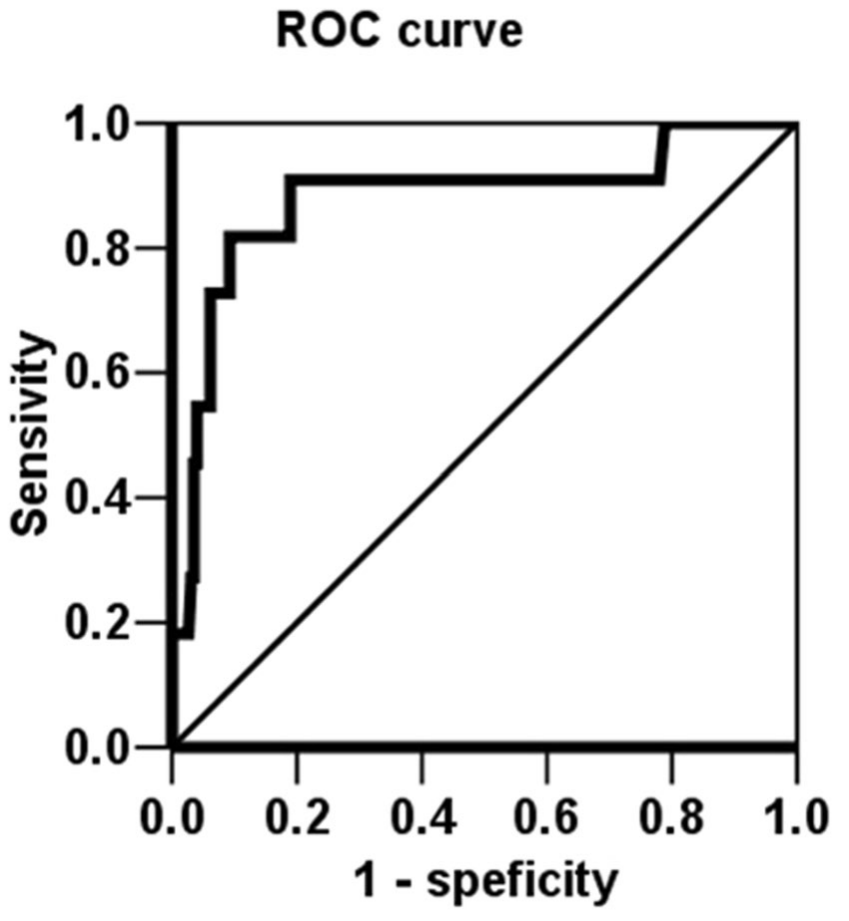
PT length is﻿ valid for predicting RSU. Results of receiver operating characteristic curve analysis are shown to identify a cutoff for using procedure times (PTs) to predict radiation-induced skin ulceration (RSU). The analysis indicates favo﻿urable predictive performance of PT for RSU events (area under curve = 0.88; p < 0.001). For predicting RSU, a cut-off PT value of 130 minutes had a sensitivity of 0.91 and a specificity of 0.81.

Univariate logistic regression analysis indicated that RSU events were significantly associated with a history of retrograde PCI (p = 0.005); use of large LAO views (p < 0.001); and PTs of >1.5 hours (p = 0.008), >2 hours (p = 0.001), >2.5 hours (p < 0.001), >3 hours (p < 0.001), >3.5 hours (p < 0.001), and >4 hours (p = 0.007); and an estimated fluoroscopy time of >1.5 hours (p < 0.001) (Table 3). The receiver operating characteristic (ROC) curve analysis showed that PT length was an accurate predictor of RSU events (area under curve = 0.88; p < 0.001) at a cut-off time of 130 minutes (sensitivity = 0.91, specificity = 0.81) (Fig. 4).

**Table 3:** Univariate Logistic Regression Analysis For Estimating Risk of RSU

Variables	Odds ratio	p value
Diabetes mellitus	2.9 (0.8–10.1)	0.090
Dyslipidemia	3.9 (0.8–18.3)	0.070
CTO PCI at non-LAD	—^a^	<0.001
Retrograde PCI technique	6.6 (1.8–24.8)	0.005
Use of a main large LAO view	—^b^	<0.001
Fluoroscopy time^c^ >1.5 hours	5.5 (2.8–155.8)	<0.001
PT > 1 hours	5.9 (0.7–46.7)	0.094
PT > 1.5 hours	16.7 (2.1–132.8)	0.008
PT > 2 hours	31.3 (3.9–249.8)	0.001
PT > 2.5 hours	30.7 (6.3–149.3)	<0.001
PT > 3 hours	35.2 (8.5–145.2)	<0.001
PT > 3.5 hours	20.2 (5.2–78.7)	<0.001
PT > 4 hours	12.4 (2.0–76.7)	0.007
Technical success^d^	0.3 (0.1–1.1)	0.071

RSU = radiation skin ulcer; CTO = chronic total occlusion; PCI = percutaneous coronary intervention; LAD = left anterior descending artery; LAO = left anterior oblique; PT = procedure time.

^a^All cases with RSU received non-LAD CTO PCIs.

^b^All cases with RSU received CTO PCI using large LAO views.

^c^A fluoroscopy time was estimated for each index CTO PCI.

^d^Completion of implantation of coronary stent(s).

## Discussion

The four main findings of this study were (1) although RSU events are often overlooked, they are severe skin complications, and the RSU events generally occurred several weeks or months after prolonged CTO PCIs; (2) all RSUs located on the right upper back were refractory to dermatological therapies and required plastic surgery; (3) a prolonged PCI performed in a fixed large LAO angle view for a non-LAD CTO lesion increased the risk of an RSU; (4) a PT of ≥ 130 minutes is an accurate predictor of a RSU event, particularly when fixed large LAO views are used.

This study is the first to recruit patients with RSU events to explore the relationship between PCI-related variables and RSU events. We previously emphasized the overlooked RSU events and presented their clinical pictures^13^. The RSU events always occurred a few weeks or months after prolonged PCIs. Iatrogenic skin complications are usually overlooked and underreported by physicians because the skin lesions do not become apparent until months after the PCIs^6, 13, 14^. A meta-analysis of 2857 CTO PCI procedures analysed from 65 studies reported only three cases with radiation-induced skin injury^6^. Another review of 26 pooled studies possibly underestimated the occurrence of radiation-induced skin injury as only 0.5% of radiation dermatitis in 3482 patients who received retrograde CTO PCIs^5^. Our data indicated that the incidence of RSU events was higher (4.6%) than that in the pooled studies. The possible reasons of the relatively higher RSU rate in our study were (1) previous underdiagnosis and underreporting; (2) referrals and educational demonstrations of very complex CTO cases in the centre; (3) inadequate skin protection; and (4) inappropriate skin biopsy and traditional treatment. All patients with RSUs observed in the study needed plastic surgery, as reported in a three-case report^8^. The poor outcomes of the RSUs suggest that coronary interventionalists must minimise RSU complications when revascularising coronary arteries.

**Figure 5:**
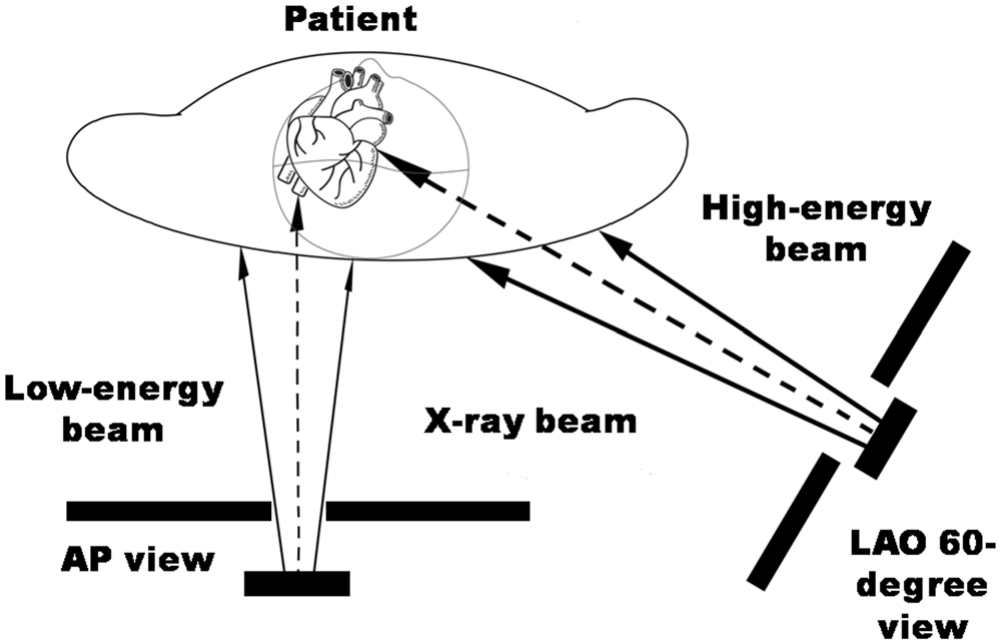
A proposed mechanism of a RSU using large left anterior oblique (LAO) views is illustrated﻿﻿﻿.﻿ Skin at right upper back exposes to high-energy beam in large LAO views in a supine patient, and in contrast, skin at middle back exposes to low-energy beam in anteroposterior (AP) views. Therefore, RSU frequently occurs in the area of right upper back.

Notably, all RSUs occurred on the right upper back (or the right parascapular area). This finding indicated that various radiation doses that irradiated different skin sites had different effects, and the doses accumulated on the right upper back were relatively high. It also implied that high-energy irradiation by X-ray emitters with large LAO angles (for example, LAO 60°) on the right upper back might result in rapid accumulation of the local radiation dose and might potentiate an RSU event. The present results clearly reveal a strong association between RSU events and large LAO views. Because a specific angiographic projection increases the distance between the irradiated local skin and the coronary arteries in these views, computerised X-ray tubes may automatically enhance the energy output and reduce the distance between the emitter and skin so that tissue penetration is sufficient to obtain acceptable images (Fig. 5). Similarly, the radiation beam energy may be automatically enhanced when obese patients receive X-ray examinations^14–16^. In brief, the use of fixed large LAO views with high-energy irradiation for long durations increases the risks of radiation-induced skin injuries compared with those of other angiographic views with low-energy irradiation. Similar results obtained from a study showed that the maximal radiation dose on skin was higher with RCA or LCX PCIs than with LAD PCIs^11^.

Determining a threshold dose of radiation exposure dose for predicting RSU events is extremely difficult because the threshold dose is affected by numerous factors. Several studies have reported that a CTO PCI compared with a non-CTO PCI is a predictor for increasing radiation skin dose^2, 3, 7, 15, 16^ and maximal entrance skin dose (ESD)^7^. Nonetheless, no standard has been proposed for accurately estimating the radiation ESD. The factors affecting radiation dose include tube voltage, collimation size, focus-to-skin distance, catheter table position, angiographic angle, and body size^3, 7, 9, 11–16^. A study reported that real-time monitoring of the dose distribution at focal skin areas enabled operators to identify high-dose skin areas^16^. Unfortunately, real-time monitoring is not widely available, and current X-ray machines are not equipped with real-time monitoring modules. Therefore, the present study proposes that a PT length exceeding 2 hours can be only considered an alternative by coronary interventionalists to minimise iatrogenic skin injury.

Data from the present study verified that PT length may be used as an alternative to real-time quantification for predicting RSU events. PT length is a valid means to accurately predict RSU events with a cut-off threshold of 130 minutes, particularly when interventionalists perform non-LAD PCIs by using fixed large LAO views. The data recommend adjustment of the beam angulations to control the ESD in case of prolonged procedures^9^. Of the RSU patients in the present study, most had PTs exceeding 3 hours (8/11, 72.7%). Therefore, interventionalists should reevaluate the net benefit when the PTs with fixed large LAO views exceed approximately 2 hours. In addition, coronary interventionalists should consider terminating the procedure early, changing the beam angulations, using a radiation filter, or modifying the therapeutic strategy to reduce the radiation dose and prevent RSU complications.

The susceptibility of patients to radiation may contribute to the occurrence of RSU events. All RSU patients were male patients who received prolonged non-LAD CTO PCIs with large LAO views. Most of them were overweight or obese (body mass index, BMI > 25 kg/m^2^). A large-scale study involving 1824 patients who received PCIs revealed that the cumulative skin dose was significantly associated with the BMI, sex, lesion complexity and location, and performing physician^12^. Taken together, the data from the present study suggest that the factors non-LAD CTO PCI, use of fixed large LAO views, retrograde technique, and PT >2–3 hours increase the risk of RSU events. Routine dermatological monitoring may be necessary after prolonged CTO PCIs and high radiation exposure doses, particularly those performed using large LAO views.

## Conclusions

The data suggest that all RSUs occurred on the right upper back. In addition, a PT length exceeding approximately 2 hours is an accurate predictor of RSU events in patients who receive prolonged non-LAD CTO PCIs with fixed large LAO projections. This study suggests that coronary interventionalists should limit the length of a PT to 2–3 hours with fixed large LAO projections to minimise RSU complications.

## Limitations

Some limitations of this study must be highlighted here. First, a small number of patients with RSUs were identified in a single cardiovascular centre. The present results may not be completely generalisable to patients treated under different laboratory and angiographic settings. Second, interventionalist- or patient-orientated referral is not ideal for selecting patients with RSUs. The incidence of other types of radiation-induced skin injuries may be ignored and underreported. Third, real-time quantification and standardisation of ESD to determine the threshold radiation skin dose for predicting an RSU event is difficult. Therefore, a PT length >2 hours was suggested as a simple alternative to real-time quantification. Finally, some factors that can potentially affect the radiation exposure dose and RSU occurrence rate, including lesion characteristics, angiographic parameters, and more detailed PCI procedures, were not investigated.

## Methods

A single-centre observational study was designed to investigate RSU events and their risk factors in patients who had received at least one CTO PCI between 1 January 2012, and December 31, 2013. We attempted to develop a strategy to prevent severe iatrogenic skin complications during complex PCIs. The study, which was conducted in collaboration with cardiologists, dermatologists, and plastic surgeons in a hospital, complied with the ethical requirements of the Declaration of Helsinki and local regulations. The medical ethics committee of the hospital approved the study protocol (VGHKS 15-CT3-08).

We selected consecutive patients who had received PCI for at least one coronary CTO lesion during the 2-year study period. A CTO lesion was defined as a totally occluded lesion identified at least 3 months before the index PCI. Index CTO PCIs involving PCIs for non-CTO lesions were also included in the study. The exclusion criteria were treatment by using a coronary artery bypass graft, PCI-related cardiovascular complications such as coronary artery perforation or tear and cardiac tamponade requiring urgent management, all-cause death during the procedure and follow-up period, or loss to follow-up for more than 6 months. Data regarding baseline characteristics, RSU management, and PCI-related parameters were collected.

In addition, CTO PCI procedures were performed at a single cardiovascular centre that had a high annual volume of PCI procedures (more than 1000 cases per year) and a certification from a national committee. The angiography system used for all procedures was an Allula Xper FD9 (Philips Medical Systems, Cleveland, Ohio). The PCIs were generally performed using a digital cine X-ray system at an acquisition rate of 15 frames per second with pulsed fluoroscopy at 30 pulses per second. An index PCI was defined as any PCI for a CTO lesion during the 2-year period of the study. The PCI-related data obtained from the computerised database at the catheterisation laboratory included approach sites and strategies, guiding catheter systems, antegrade and/or retrograde techniques, main angiographic angulations during PCI, PT, and technical successes or failure. A PT was defined as the interval between the times of the first and last angiograms in each index PCI. The main angiographic view indicated that the angiographic angulation in the specified view was mainly used for performing the CTO PCI. All PCI strategies, techniques, and angiographic angles were used according to the discretion of each individual interventionalist. All study-related procedures were performed by board-certified coronary interventionalists with 5 to 30 years of experience. To minimise interobserver bias, retrospective interpretations of the angiographic findings were independently obtained from two experienced physicians.

Patients who had developed skin lesions after undergoing the index CTO PCIs were initially assessed by cardiologists and then referred to dermatologists in the same hospital for further management. The referral criteria for suspected radiation-induced skin injury on the back were (1) rash or erythema, (2) refractory skin itching or pain, or (3) skin ulceration. Once radiation-induced dermatitis was impressed by a dermatologist within 6 months after the index CTO PCI, the patients with skin lesions were enrolled as the participants for treatments and scheduled follow-ups. Each skin lesion was reviewed and diagnosed by two independent and experienced dermatologists who identified radiation-induced skin lesions. In case of a disagreement between the dermatologists, the lesion would be further classified according to a dermatological committee. Biopsies for pathological assessments and diagnosis were performed according to the discretion of the managing dermatologist. Patients who received a diagnosis of skin lesions other than RSUs, including radiation-induced dermatitis, contact dermatitis, morphea, and fixed drug eruption, were excluded. A radiation-induced skin lesion might progress to an ulcerative wound with a poor-healing appearance, which was considered an RSU event. The RSU lesions were periodically assessed by the dermatologists or plastic surgeons in the hospital during the 2-year follow-up. Intractable RSU wounds were treated through plastic surgery, including debridement, skin graft, or flap reconstruction. The analysis excluded patients who died of any cause or who were lost to follow-up for more than 6 months.

### Statistical analysis

All variables were analysed using SPSS for Windows (SPSS Inc., Chicago, Illinois, USA). All categorical data and rates were represented as percentages and numbers, and continuous data were presented as means ± standard deviations. Baseline characteristics and PCI data were compared between patients with and without RSU by using a chi-square test or a Fisher exact test for categorical variables, independent *t*-tests for continuous variables, and a Mann-Whitney *U*-test for non-normally distributed continuous variables. ROC curve analysis was used to identify cut-off values for using PT to predict the occurrence of RSU. A value of p < 0.05 with a two-sided 95% confidence interval was considered statistically significant for all tests.
